# Employees’ Perspectives on the Facilitators and Barriers to Engaging With Digital Mental Health Interventions in the Workplace: Qualitative Study

**DOI:** 10.2196/mental.9146

**Published:** 2018-01-19

**Authors:** Stephany Carolan, Richard O de Visser

**Affiliations:** ^1^ School of Psychology University of Sussex Brighton United Kingdom

**Keywords:** anxiety, depression, eHealth, Internet, mental health, mHealth, occupational, online, stress, workplace

## Abstract

**Background:**

Prevalence rates of work-related stress, depression, and anxiety are high, resulting in reduced productivity and increased absenteeism. There is evidence that these conditions can be successfully treated in the workplace, but take-up of psychological treatments among workers is low. Digital mental health interventions delivered in the workplace may be one way to address this imbalance, but although there is evidence that digital mental health is effective at treating stress, depression, and anxiety in the workplace, uptake of and engagement with these interventions remains a concern. Additionally, there is little research on the appropriateness of the workplace for delivering these interventions or on what the facilitators and barriers to engagement with digital mental health interventions in an occupational setting might be.

**Objective:**

The aim of this research was to get a better understanding of the facilitators and barriers to engaging with digital mental health interventions in the workplace.

**Methods:**

Semistructured interviews were held with 18 participants who had access to an occupational digital mental health intervention as part of a randomized controlled trial. The interviews were transcribed, and thematic analysis was used to develop an understanding of the data.

**Results:**

Digital mental health interventions were described by interviewees as convenient, flexible, and anonymous; these attributes were seen as being both facilitators and barriers to engagement in a workplace setting. Convenience and flexibility could increase the opportunities to engage with digital mental health, but in a workplace setting they could also result in difficulty in prioritizing time and ensuring a temporal and spatial separation between work and therapy. The anonymity of the Internet could encourage use, but that benefit may be lost for people who work in open-plan offices. Other facilitators to engagement included interactive and interesting content and design features such as progress trackers and reminders to log in. The main barrier to engagement was the lack of time. The perfect digital mental health intervention was described as a website that combined a short interactive course that was accessed alongside time-unlimited information and advice that was regularly updated and could be dipped in and out of. Participants also wanted access to e-coaching support.

**Conclusions:**

Occupational digital mental health interventions may have an important role in delivering health care support to employees. Although the advantages of digital mental health interventions are clear, they do not always fully translate to interventions delivered in an occupational setting and further work is required to identify ways of minimizing potential barriers to access and engagement.

**Trial Registration:**

ClinicalTrials.gov: NCT02729987; https://clinicaltrials.gov/ct2/show/NCT02729987?term=NCT02729987& rank=1 (Archived at WebCite at http://www.webcitation.org/6wZJge9rt)

## Introduction

### Background

Nearly 1 in 3 workers in Europe report that they are affected by work-related stress, which is estimated to cost between 3% and 4% of gross national product [1]. Along with a societal and individual cost, common mental health problems such as stress, depression, and anxiety have a cost to organizations. They are associated with reduced productivity [2-5], early retirement [6], increased sickness absence [7-8], presenteeism (not working at capacity while at work) [9], and staff turnover through health-related job loss [10]. There is evidence that these conditions can be successfully prevented and treated in the workplace [11-14], but take-up of psychological treatments among workers is low, resulting in many workers going untreated [2,15,16]. One way of increasing workers’ access to psychological treatments might be through the use of digital mental health interventions in the workplace. A recent meta-analysis found that these interventions are effective in increasing psychological well-being and workplace effectiveness but that the mean intervention completion (the extent to which participants adhered to the intervention) was 45%, with a range of 3% to 95% [17]. Although there are examples of occupational digital mental health interventions that have achieved good adherence [18-21], uptake of and engagement with these interventions in the workplace clearly remains a pressing concern.

Researchers cite a number of advantages to digital health interventions compared with traditional face-to-face interventions: these are often described as the anonymity and accessibility of the Internet with clients being able to access treatment at a time, a place, and at a pace that is convenient to them [22-24]. These advantages have led digital health interventions to being described as being well suited for the workplace [25], but with occupational digital mental health interventions still being in their infancy, little research has been done to see if these perceived advantages translate to an occupational setting; furthermore, little research has been done on the barriers and facilitators to take up and engagement with digital health interventions in a workplace setting.

The study reported here used qualitative interviews to increase understanding of the experiences of participants using an occupational digital mental health intervention as part of a randomized controlled trial (RCT). Combining quantitative and qualitative data is recommended as an effective means of getting a better understanding of new and innovative technologies [26] and other interventions [27].

The RCT compared access to a Web-based stress management intervention (WorkGuru) with and without access to an online facilitated discussion group. Full details of the trial are reported elsewhere [28,29]. WorkGuru is an 8-week modular program that is based on the principles of cognitive behavioral therapy (CBT), positive psychology, mindfulness, and problem solving. The intervention can be accessed on a secure platform on a computer or smartphone. There are 7 core modules and 3 optional modules. People completed the modules in the order and at a pace that they chose. The modules consisted of educational reading, interactive exercises, a stress and a thought diary, audio, and short animations. Participants could choose to share their work with an e-coach and could contact the coach for information or advice. The coach responded within 24 hours. The e-coach contacted each participant 3 times during the course of the 8-week program with reminders to login. Participants could also choose to opt-in to automated reminders (sent at a time and frequency that they chose) and a motivational message sent every Monday (the Monday morning message). Both reminders were sent by email. Along with the modules, participants could complete 8 self-monitoring standardized questionnaires.

The original trial population was recruited from 6 UK-based organizations: 2 local authorities, 2 universities, 1 third sector (not for profit) organization, and 1 telecommunication organization. Participants in the trial were randomized to 1 of 3 groups: the minimal support group (accessing the intervention with minimal support from an e-coach), the discussion group (access to the intervention with minimal support from an e-coach plus an online facilitated learning group), or the control group (access to the intervention after follow-up). Eligibility criteria for the RCT were as follows: (1) aged 18 years or over, (2) employed by a participating organization, (3) willing to engage with a digital CBT-based stress management intervention, (4) access to the Internet, (5) access to a tablet or computer, and (6) an elevated level of stress as demonstrated by a score of ≥20 on the ten-item Perceived Stress Scale (PSS-10) [30].

### Research Questions

The research questions for this study were as follows: (1) What did participants see as the positives and the negatives of occupational digital mental health? (2) What helped and what hindered engagement with occupational digital mental health? (3) What more could be done to help participants engage with occupational digital mental health? (4) What did participants think a perfect digital mental health intervention would look like?

## Methods

### Participants

All participants (n=82) recruited to the RCT were invited via email to take part in this study. Four emails were sent over a 3-week period, inviting participation in telephone interviews. Further information about the study was given. The emails emphasized that we were keen to interview participants whether or not they had logged on to the program. The final email re-emphasized our wish to interview participants who had not engaged with the program. Participants were invited to contact the first author for more information and to arrange a time for the interview. Informed consent forms were distributed and returned before the interview. Ethical approval was granted by the host university’s ethics committee.

### Data Collection

A total of 18 semistructured telephone interviews were conducted by the first author in May 2017. Each interview lasted between 20 and 50 min. The interview questions were informed by previous literature, experience from the RCT, and the study aims. The final question used a solutions focus approach (see [31]) to invite participants to imagine a perfect occupational digital mental health intervention. Participants received and were asked to read a participant information sheet informing them about the study, and they were asked to sign and return a consent form or give audio-recorded informed consent before the interview takes place. Interview recordings were transcribed verbatim and anonymized.

### Data Analysis

Thematic analysis as described by Braun and Clarke [32] was used to develop an understanding of the data. The 6 phases of thematic analysis described by Braun and Clarke [32] are as follows: (1) familiarize yourself with the data, (2) generate initial codes, (3) search for themes, (4) review themes, (5) define and name themes, and (6) produce the report. Microsoft Excel (2011) was used to organize and manage the data. Both authors independently reviewed and coded a subset of the transcripts and discussed and resolved any inconsistencies to arrive at a shared interpretation of the data. The first author coded the remaining transcripts, which were reviewed by the second author for inconsistencies. Identifier pseudonyms were used.

## Results

### Recruitment and Participants

A comparison between the study participants and the original trial participants is given in Table 1. All participants were white. The sample was, on average, older (45 years compared with 41 years) and included less female participants (78% compared with 85%) than the original study. Recruitment from the universities and the telecommunication organization was broadly similar, but more participants were recruited from the third sector organization, and we were not able to recruit any participants from the 2 local authorities. The number of people in this study who recalled being randomized to the control group was representative of the original study, but the number that recalled that they had been randomized to the minimal support group was higher, and to the discussion group lower. Of the 18 participants in this study, 14 respondents (78%) reported that their work was predominantly office based; the remaining 4 (22%) reported a mixture of office and client work.

When participants were asked whether they thought they had engaged well with the intervention, 7 (39%) said they had engaged well, 8 said no or not very well (44%), and 3 had never logged into the intervention (17%). Participants were also asked to recall how many times they had logged into the program. The mean number of logins recalled by participants who said that they had engaged well with the intervention was 15.0 (range 4-30); the mean number for those who recalled that they had not engaged well was 9.8 (range 5-20).

All participants who accessed WorkGuru did so during working hours (including their lunch break), with only 2 saying that they also accessed it outside working hours. The initial trigger for accessing the intervention was described as current experience of stress, with a number of participants saying that the opportunity to use it arose at the right time. Participants said that they were looking for tools to help them cope with their stress. Moreover, 14 (78%) of the people interviewed for this study said that they had never used a digital health intervention before using WorkGuru. Of the remaining 4 participants, 3 had used a pedometer, 1 used a mood tracker, 1 monitored his or her sleep, and 1 participant accessed YouTube videos designed to help people sleep.

A total of 6 key themes were derived from the analysis: the positives and negatives of digital mental health; the facilitators and barriers to engagement; the role of the e-coach; and what made a perfect occupational digital health intervention.

**Table 1:** Comparison of participants in this study and the original trial. RCT: randomized control trial.

Comparison variable		Participants in this study (N=18)	Participants in RCT (N=82)
Mean age (SD)		45 (10.8)	41 (10.2)
Female, n (%)		14 (78)	70 (85)
**Organization, n (%)**			
	Third sector	7 (39)	17 (21)
	Universities	10 (56)	48 (58)
	Telecommunications	1 (5)	3 (4)
	Local authority	0 (0)	14 (17)
**Allocated group, n (%)**			
	Discussion group	4 (22)	26 (32)
	Minimal support group	8 (44)	28 (34)
	Wait list control	6 (33)	28 (34)

### The Positives of Digital Mental Health Interventions

Participants described digital mental health interventions as being convenient both in terms of accessing it at a time that is convenient for them and at a place that is convenient for them. The quote below reflects participants’ appreciation of these characteristics:

Whenever I need something I can just straight away go there without waiting for someone, waiting for an appointment or like. I can get help as soon as possible and I can get it anywhere because it’s online on the Internet.

Sara, 31 years, university one

Another aspect of this convenience identified by participants was the ability to work at a time that was convenient to them. Natalie [40 years, third sector] noted that the intervention gave “flexibility to access the intervention at a time that you can fit into your work diary.” This meant that they could fit sessions in when they had time rather than having to fit with the timetable of a (potentially busy) therapist. Robert also appreciated the flexibility of access and talked about the importance of being able to work at his own pace:

It’s incredibly accessible both in terms that I could choose when I was engaging with it, and it allowed me therefore to kind of pace myself and reflect on things and then go back to things when I wanted to rather than saying: “Well you’ve got a session, it’s at 2 o’clock on a Friday and that’s it, that’s your only window”. So I think it made it in some senses more live for me rather than an event that you go to.

Robert, 46 years, university one

Participants identified the stigma of mental illness as still being an issue in the workplace. One participant said:

I wouldn’t tell it to anyone in my workplace.

Sara, 31 years, university one

Another participant described how she would not talk to her employer about the elements of work that contributed to her stress as:

I would then be forever seen as someone who doesn’t cope well and then wouldn’t get much career progression

Sue, 43 years, university two

Participants suggested that the discreteness and anonymity of digital mental health interventions helped them to overcome their fear of the stigma:

I think also it’s very discreet. If you have to shuffle off and actually see somebody you know face to face, it’s a bit more public, people are more likely to know about it.

Fiona, 62 years, third sector

The privacy of the Internet allowed participants to access support without work colleagues knowing. For example, Simon [48 years, university two] noted that the intervention allowed him to “get the support without necessarily drawing attention to myself at work. ” Anonymity was also given by not having to call someone to make an appointment:

Personally it was easier to say, “I’m doing something to help myself,” but without actually having to speak to someone. You know it’s quite daunting if you’ve got a worry to actually pick up the phone and speak to someone.

Anna, 47 years, third sector

Anna found it easier to start the digital intervention because she did not have to speak to someone to make an appointment; other participants shared this view and suggested that by having access to a Web-based intervention they were able to access treatment, which they might not have done if they had to speak face-to-face with someone:

I felt quite positive about starting it off when it’s not something I would’ve done if I’d had to go and physically speak to somebody about it.

Tony, 56 years, third sector

Some participants valued being able to access the intervention in the workplace. This feature enabled them take time out of stressful events at work to focus on themselves:

To be able to in a workplace setting after dealing with a particularly stressful case, being able to remove yourself and do something just for you with permission from your employer, was really an empowering tool that they gave us.

Jane, 28 years, third sector

Jane valued being able to access the intervention in the workplace, but other participants identified a number of barriers to accessing digital mental health interventions at work; these are described in the next section.

### The Negatives of Digital Mental Health Interventions

Participants identified a number of negatives to accessing digital interventions in the workplace. These included not having a defined time in which to use the intervention. Although participants appreciated the flexibility of digital mental health interventions, a number of them also felt that they needed more self-discipline to remain engaged with a digital intervention compared with a face-to-face intervention where they had an appointment in their diary and an office or clinic to visit:

It’s good not to have to do things in a certain time but it’s also not good because you can often think “Actually I’ll do it later,” and never get round to it. [...] If it’s online it’s down to the individual themselves to go and do what they are required to do.

Simon, 48 years, university two

Other participants struggled with not having a private space to access the intervention:

And the other problem is sitting in an open plan, hot-desking space. In our room each desk runs into the next desk, there are no privacy screens between them. So I don’t know if there’s a sense of feeling that other colleagues can see what you’re working on, they can see the screen of your computer.

Natalie, 40 years, third sector

For some participants, accessing the intervention at their desks meant that they might have benefited less from it, because existing ongoing work concerns that may have been the *cause* of stress were present in the therapeutic environment:

If you go somewhere else to an appointment, I think on the whole you’re going to get more out of it than if you’re fitting it in but you’re still at your desk and you can see the invoices that need approving and your to-do list.

Katy, 63 years, university one

In addition to the lack of a *spatial* separation between work and therapy, there was also no *temporal* separation between work and therapy. For example, one interviewee noted that accessing the digital intervention at her desk meant that she did not have the journey back to work to help her switch back to work mode:

You’re doing something very reflective and personal that might make you feel uncomfortable feelings, and then to go back into work mode immediately. I guess I think even if you go to a counselling session you have that physical journey back to work which helps switch modes back and so you’ve got time to kind of leave those feelings behind.

Sue, 43 years, university two

Another issue was that the workplace is often a place in which we are invested in appearing strong and capable. For example, one participant described how, although she was able to present herself positively to work colleagues, reflecting on her mental health in the workplace left her feeling exposed:

I was struggling. At work people probably wouldn’t really have picked up that much was going on for me, I was quite happy to keep that going in front of people so then I’m at work and I’m...it starts you having to think about the other stuff that’s affecting you internally but you’re managing to put on a pretty OK persona when you’re at work so then it just felt like I was having to...I didn’t want to expose myself too much I suppose.

Anna, 47 years, third sector

Several participants said that one of the problems for them with completing a minimally guided digital intervention was the lack of human interaction. Although not having to speak to someone was a positive for some people (see above), it also meant that it was easier to disengage from the intervention:

It does allow you to maybe explore these things without having to open up directly to a person. But then the downside to that is that it also allows you to walk away from it more easily.

Tony, 56 years, third sector

Some participants noted that not having a one-to-one interaction meant that they might choose the “easier” elements of the intervention, and therefore not obtain the benefits of more comprehensive engagement. For example, John [33 years, university two] noted that it was possible to avoid the more challenging elements that “probably had more growth behind it *.* ” The lack of face-to-face contact also meant that participants could be left feeling isolated and feeling that they had not made an emotional connection and that they were not “ *sharing* ”:

I guess it’s the isolation, with doing everything anonymously and just taking time out on your own to do it there’s no real sharing involved in it

Jane, 28 years, third sector

### Facilitators to Engagement

In addition to the convenience, flexibility, and anonymity mentioned above, the main factors that participants identified as helping them to engage with the digital intervention were program content and design. Interesting content was one reason given for engaging with the program. For example, 1 participant said:

The content I think was what kept me going back into it because it was interesting. It had interesting content.

John, 33 years, university two

Participants liked that the program was interactive and they liked the way it was presented. The positive experience motivated them to continue:

It was in nice bite size chunks. It was well presented. It was quite enjoyable. Yeah, it was quite enjoyable to do. It was good taking yourself out of the work situation for a bit, before going back in again. So I mean it was just a very positive experience so I think that just encouraged me to carry on with it.

Claire, 57 years, university one

Each module gave an estimation of the amount of time it would take to complete, which enabled users to plan their engagement. Participants also appreciated that the intervention tracked their progress through the program; for example, 1 participant described how being able to see what modules she had completed motivated her to compete other modules:

You can see on screen you’ve done this and you’ve done this and you’ve done this, but you still need to do this. It was almost like playing an online game.

Katy, 63 years, university one

Other features that helped participants to engage with the intervention were reminders to log in that were built into the system. These included self-timed opt-in automated emails and the opt-in Monday morning message. This was an email message sent every Monday morning that included a motivational message and information on keeping yourself psychologically well at work. It was intended as a reinforcement of the key messages in the program and a reminder to log in. Personalized reminders were also provided by the e-coach who contacted each participant to remind them to log in to the program and to contact her if they needed any support. One participant suggested that email reminders from the e-coach were more helpful than the automated reminders:

I think when I got the emails from the work coach themselves, because it was a person enquiring that was much more of a prompt to look in and go: “Oh yeah, gosh, I do need to focus in on this and make some time for it,” but when it was just an automated response it kind of felt, it kind of made me feel guilty about logging in.

Jane, 28 years, third sector

In addition to using the different reminders within the intervention, some participants described setting their own reminders by putting tasks in their work calendar. They noted that this helped them to engage with the program:

If you just think you’ve got forever to do it, it would have been easier to put it off whereas you know I wanted to do it so I set myself reminders and built it into my calendar.

Claire, 57 years, university one

A number of participants identified the importance of organizations and line managers in promoting the use of interventions such as WorkGuru and encouraging the staff to use them. Natalie described how support to use the intervention from a manager could make a big difference:

If you get a message from the manager that that’s ok and that they encourage and support you to do that, that can make a big difference.

Natalie, 40 years, third sector

Promotion by the employer gave the intervention legitimacy and gave the staff explicit permission to use it:

I think probably the fact that this was circulated by the university, it probably added a bit of...almost legitimacy about it, I guess. This was something that was supported by the university, which is probably a little bit silly but when you’re in a stressed situation it is just the knowledge that yeah well the university said this is an ok thing to do, it’s ok for me to take time to be working through this and it’s to their benefit because if I’m working more effectively then they benefit as well.

Claire, 57 years, university one

### Barriers to Engagement

Over half of the interviewees identified the pressure of time or excessive workload as being the main reasons for not engaging with the intervention:

Although it was something that I wanted to do, getting [the prompt to logon] was just kind of a: “Oh god, have I really got time to do this today? Am I going to feel guilty for leaving my colleagues?”

Jane, 28 years, third sector

Similarly, Anna [47 years, third sector] noted that engaging with the intervention *“became almost a luxury”*, and that when work pressures were mounting *“I couldn’t devote the time to do it."*

In addition to time pressure, the symptoms of mental health problems were identified as potential barriers to engagement. For example, Chloe noted that effective engagement required levels of motivation that may not be possessed by people with depression:

Probably at the time, um I was very low, very depressed. [...] I suppose time would’ve been a bit of an issue, coupled with depression. I didn’t have any motivation at all.

Chloe, 44 years, telecommunication

### The Role of the E-Coach

Participants gave mixed reports on their use, appreciation, and expectation of the e-coach. A number of participants did not engage with the e-coach; some were unclear about what the role of the e-coach was or how they could use her support:

I thought it really helped when I did some of these exercises and like sitting and writing down the feelings that could happen or triggers. I did it a couple of times and it really helped me a lot so I don’t know how to tell it to the coach. Can the coach help with this stuff or not? Also in the exercises they are there and what else can the coach help with?

Sara, 31 years, university one

One participant said that the communication from the e-coach felt automated:

Yeah it just, it seemed like an automated thing. I didn’t really, I mean obviously I thought if you sent them an email it would get through to someone but um it just didn’t feel very personal I guess.

Rose, 38 years, university one

However, another participant had a more positive experience:

I actually found the initial contact, really really, almost like validating. I was an individual I wasn’t just a number, which I kind of really, really...really impressed me.

Robert, 46 years, university one

Participants were also divided about how proactive they wanted the e-coach to be. Some participants were happy that the e-coach was there if they wanted to ask any questions or *“if I’ve got a specific query”* [Claire, 57 years, university one].

Other participants wanted more contact with the e-coach:

I think it would be useful to have something a bit more proactive near the front just to try and ensure people really were comfortable with what they were doing.

Tony, 56 years, third sector

### What Would a Perfect Digital Intervention Look Like?

When asked to describe what a perfect occupational digital mental health intervention might look like, almost half of the interviewees said that they would want to be able to access it only on a computer, the same number said on both a computer and a smartphone, and 2 said they would like to access the intervention only on a smartphone.

Participants wanted an intervention that would be anonymous and confidential and that could be tailored or adapted so that it could meet the needs of different people:

It’s just remembering that everyone is different and everyone’s moods has ups and downs, and depressions and joys are addressed in different ways and I guess a single program that takes everyone through a singular route probably doesn’t hit the nail on the head.

Tony, 56 years, third sector

Nearly all participants described their perfect intervention as combining a short course that they could work through independently with a website that had regularly updated information and personalized advice that they could make use of as required over an indefinite period:

It would be sort of as I described, a short, fairly intensive course that you were checked up on whether you’d done it or not which would really help followed by the availability continuously after that, um, just for dipping into or for necessarily contacting somebody in person if possible.

Rachel, 55 years, university one

Interviewees said that the structure and layout of the short course should be simple, especially those who were less confident using information technology:

Yeah and it has got to be something very simple because I’m really not very technical. I am a bit of a, yeah a technology dinosaur to be honest so it would have to be very simple and accessible.

Natalie, 40 years, third sector

They also suggested that the content of the course should be interactive and consist of a mixture of reading and listening:

It’s got to be something like this [WorkGuru] ...for me anyway, something that is interactive...because that’s how I engage with stuff, it can’t be just reading. I like that this was a mixture of reading, listening and actually doing stuff because I think it would be very easy not to take it in if it was just reading from a screen.

Claire, 57 years, university one

Participants were equally split between those wanting peer support as part of the time-unlimited resource and those who did not. One participant suggested that if peer support was available, she would want a small group:

If it was going to be something that I use regularly then I would probably want a smaller peer group, as in the sort of size that was in the discussion group that was active with WorkGuru rather than it being a kind of Facebook type thing where anybody can get involved because I think that floods it, and it becomes too much to actually digest and get involved with.

Jill, 31 years, third sector

In contrast, Rose stated that she would not use a support group for the following reason:

I’m not good with groups of people really so that’s not something I’d make much use of myself.

Rose, 38 years, university one

A number of participants suggested that monitoring, including self-report tracking of stress symptoms, would be useful but emphasized that this information should not be made available to their employer.

The majority of participants wanted to be able to contact a coach if needed. For some, that support could be asynchronistic, but others wanted live chat either through video (eg, Skype) or instant messaging. A participant said:

You kind of sense the difference between someone who is physically there the whole time and yeah they’re there, they’re writing an answer but it’s like an email conversation.

John, 33 years, university two

## Discussion

### Engagement With the Intervention

Only 4 interviewees said that they had used a digital health intervention before using WorkGuru. This suggests that despite the growing number of apps and websites, digital health is still a very underutilized resource. The trigger for initially accessing the intervention in this study was described by participants as a current experience of stress. This may suggest that perceived personal relevance is an important factor in initiating engagement with digital health interventions [33].

### Positives and Negatives of Digital Mental Health in the Workplace

Participants in this study described contradictions between aspects of occupational digital mental health interventions, viewing the same aspects as both advantages and disadvantages. Convenience and flexibility could increase engagement with digital mental health by increasing the opportunities to access the intervention, but within a work environment, these advantages could also be experienced as disadvantages, resulting in difficulties in prioritizing time and a lack of spatial and temporal separation between work and therapy, which left some people feeling that they had competing priorities, or left them feeling exposed as they struggled to move from therapy mode to work mode. Knowles et al [34,35] identified similar contradictions in users’ experience of digital therapies in nonwork settings. They identified contradictions in users’ experience of flexibility, support, autonomy, connectedness, and anonymity in computerized therapy for depression and anxiety delivered predominantly in primary care.

In this study, the anonymity of digital health interventions was hard to maintain within an open-plan environment. Anonymity was important because it enabled participants to access help without fear of stigma and for some people it gave them the confidence to use the intervention, which they may not have done if they had to attend a face-to-face appointment or speak to their general practitioner (GP). However, other participants suggested that anonymity made it easier to disengage from the intervention. It could be argued that by removing some of the barriers to accessing face-to-face interventions such as inconvenient locations, inability to get an appointment, high cost, lack of transport, delay in access, and the fear of stigma, digital mental health may increase the number of people that take up therapy [36], but one of the effects of easing access to interventions may be increased dropout [37]. We can draw on the Prochaska and DiClemente’s [38] stages of change model to illustrate this further. Prochaska and DiClemente ([38]; see also [39]) described 5 stages of behavioral change: (1) precontemplation (where there is no intention to change a behavior), (2) contemplation (where people are thinking about changing a behavior), (3) preparation (where people are intending to take action and may be taking small steps toward it), (4) action (where people are taking action), and (5) maintenance (where people work to prevent relapse). People who have made an appointment to attend a face-to-face intervention are more likely to be in the action stages of change, whereas people accessing digital interventions may also be in the contemplation and preparation stages of change. They may be accessing the intervention out of curiosity—a wish to explore the possibility without making a commitment. This means that they may move back to the contemplation or preparation stages of the change model and may wish to access the digital intervention or another form of psychological intervention at a later date. In widening access to therapies, digital mental health interventions may be the first step in someone’s therapeutic journey, and as such, disengagement should not necessarily be seen as a failure but as part of a process of seeking help. Our current data do not allow us to identify which users of digital mental health interventions are in which stage of the change model; future research may wish to explore this further to gain a better understanding of the role digital mental health interventions play in enabling people to access support and to change behaviors.

Along with being able to disengage from the intervention more easily, one of the other potential disadvantages of the lack of face-to-face contact in minimally supported digital mental health interventions identified by participants was the lack of emotional connection. Even when guidance is available, it is often voluntary, and users can choose not to engage with the e-coach. Some participants described feelings of isolation. An important component of traditional therapy is the therapeutic alliance, which is defined as the collaborative bond between therapist and patient [40]. Despite feelings of isolation expressed by some participants, there is evidence that a positive therapeutic alliance can develop in fully automated digital mental health interventions [41]. Clarke et al [41] found that the therapeutic alliance in a digital environment was not associated with treatment gains (in contrast to face-to-face psychotherapies), but that it was correlated with levels of engagement; perceived emotional engagement correlated positively with program use.

### Facilitators and Barriers to Engagement

Along with the convenience, flexibility, and anonymity of digital mental health interventions, participants in this study identified program content and design as a facilitator to engagement. They liked that the program was interactive and that it was presented well. Intrinsic motivation (finding the content interesting) has been shown to be an important factor in treatment adherence to digital health interventions [42], as is design and appearance [33,43,44]. If people like an intervention they are more likely to continue with it [44]. Design features appreciated by participants included estimation of time to complete each module, a progress tracker, and reminders to log in and use the intervention. There is evidence that reminders increase engagement with digital interventions [45-47] and that people who choose to receive reminders to log in and choose to receive motivational emails show greater symptom reduction [48]. There is also evidence, however, that these email prompts could be easily ignored (and even resented) in a workplace context as a consequence of a full inbox [49]. There was some evidence of this in this study, but almost half of the participants mentioned receiving and appreciating the Monday morning message; this suggests that when reminders have an additional value (ie, motivational quotes and well-being information and advice), they are more likely to stand out in a busy email inbox.

The role of the organization and line managers was identified as an important facilitator to engagement with the digital mental health intervention. It was important to many of the participants that their use of the intervention was confidential; stigma about mental illness was still something that was perceived as being prevalent in the workplace, with some participants saying that knowledge about their mental health problems could be career limiting. Research supports this perspective with evidence that the stigma associated with mental ill health can result in lower wages [50], underemployment, and precarious employment [51]. However, although participants did not necessarily want their employer to know that they were accessing the intervention, they did think that it was important for organizations and line managers to circulate information about the intervention and to encourage its use. Organizational support gave the intervention legitimacy and signaled to the employees that they could use it. By circulating this information, organizations would be showing explicit concern for employee well-being, which has been shown to result in higher levels of employee commitment to the organization [52]. Further research is needed to get a better understanding about the role of organizations in promoting take-up and engagement with occupational digital mental health interventions.

Participants identified the lack of time as the main barrier to engaging with digital mental health interventions in the workplace. The lack of time has been identified by other studies on digital health interventions delivered in the workplace as a reason given by participants for disengaging from interventions [53-56]. Future research could explore further the role of employers in helping employees to prioritize accessing digital mental health interventions in the workplace.

### The Role of the E-Coach

The intervention used in this research provided minimal guided support from an e-coach. In line with other minimal guided interventions (see [57]), the e-coach provided adherence support (log-in reminders) and feedback on request. Interviewees were divided by their experience of the e-coach and by how proactive they wanted the coaching to be. This division suggests that the type of support people want is a personal preference and might be best negotiated with the individual at the start of the program.

### The Perfect Web-Based Intervention

When describing their perfect digital mental health intervention, interviewees described a simple, interactive, and easy-to-navigate website that could be accessed via a computer or a smartphone. There are advantages to delivering interventions via mobile devices such as smartphones (eg, the ability to employ ecological momentary assessments and to deliver interventions at moments of high need), but research in this area still remains in its infancy [45,58]. It was important to interviewees that the perfect intervention was anonymous and confidential and that it could be personalized (ie, tailored to their needs). Tailored interventions have been shown to be more effective than standardized approach to delivering digital interventions [59]. The intervention would combine a short course that users could work through independently with regularly updated, time-unlimited information and advice that they could dip in and out of over a longer period. The short course described by interviewees reflects features identified in a systematic review as increasing engagement with occupational digital mental health interventions [17]; these include providing guidance, delivering in a short time frame (6-7 weeks), tailoring, and self-monitoring. Regularly updated content has been identified as an inducement to revisiting digital interventions [43]. To our knowledge, no other study on digital mental health interventions has identified the desire to access time-unlimited information and advice.

Interviewees reported that they wanted support from an e-coach but were divided about whether the support should be asynchronistic or synchronistic. Digital interventions that provide human guidance have been shown to be superior to unguided interventions [24,47,60-63], but currently there is no research comparing asynchronistic guidance with synchronistic guidance in digital mental health interventions.

A number of interviewees suggested incorporating self-monitoring, including self-report of stress symptoms. Self-monitoring is a core feature of many behavioral and psychological therapies [64] and has been recommended as an important component in the delivery of digital mental health [45]. Interviewees were divided about the use of peer support with some people saying they would like it and others saying they would not use it. There is currently little evidence to support the use of online peer support groups for people experiencing depression [65,66] or for young people experiencing mental health problems [67].

### Implications for the Workplace

The findings from this study suggest that the role of organizations and line managers is crucial to promoting the use of digital mental health interventions in the workplace. For some employees, digital mental health interventions were an important means of accessing convenient and flexible support, and it formed an important component of a broader health and well-being strategy. To encourage uptake and engagement with these interventions, organizations and line managers must actively promote the interventions, and while maintaining confidentiality, support the staff to prioritize time during working hours and identify a private space to access the intervention and to reflect on the content.

### Limitations

One of the limitations identified in the original study was that the participants recruited to the study (predominantly well-educated women working in social care or the knowledge industry in senior manager or administrative roles) were not representative of the general workforce. This limitation is evident in this study. Moreover, the majority of participants recruited in this study reported that their work was predominantly office based and all participants described having some autonomy over their work schedule. It is highly likely that the facilitators and barriers to the use of digital mental health interventions among other working groups (eg, employees working in blue-collar roles or in the service industries) will be different to those experienced by autonomous, office-based workers. There is a strong need for research into the use of occupational digital mental health interventions to be conducted in occupations and industries that are traditionally underrepresented (or wholly absent) in current studies.

Although this study was successful in engaging participants who did not perceive themselves as having engaged well with the intervention, participants were from a self-selecting group of employees who volunteered for the original trial and, therefore, did have some interest in engaging with digital mental health interventions. Therefore, we were unable to study the views of employees who may be less open to engaging with digital health interventions.

Another limitation to this study is the 1-year gap between participants being recruited to the original trial and being interviewed for this study. This meant that the study relied on participants’ recollection of their experience, which may be flawed.

### Conclusions

Occupational digital mental health interventions have an important role in delivering health care support to employees in the workplace and should form part of a broader health and well-being package. For some people, digital mental health interventions delivered in the workplace may help them to access help, which they may not have done if they had to access face-to-face therapies or speak to their GP. The convenience, flexibility, and anonymity of digital mental health interventions was experienced as both positives and as negatives, helping people to engage with occupational digital mental health, but also acting as barriers to engagement. It is important that developers of digital interventions and employers work with employees to overcome these challenges.

## Abbreviations

CBT: cognitive behavioral therapy
GP: general practitioner
PSS: Perceived Stress Scale
RCT: randomized controlled trial

## References

[1] Leka S, Jain A (2010). Apps.who.int.

[2] Birnbaum HG, Kessler RC, Kelley D, Ben-Hamadi R, Joish VN, Greenberg PE (2010). Employer burden of mild, moderate, and severe major depressive disorder: mental health services utilization and costs, and work performance. Depress Anxiety.

[3] Dewa CS, Thompson AH, Jacobs P (2011). The association of treatment of depressive episodes and work productivity. Can J Psychiatry.

[4] Gutman DA, Nemeroff CB, Contrada R, Baum A (2011). Stress and depression. The Handbook of Stress Science: Biology, Psychology, and Health.

[5] Waghorn G, Chant D, White P, Whiteford H (2005). Disability, employment and work performance among people with ICD-10 anxiety disorders. Aust N Z J Psychiatry.

[6] Karpansalo M, Kauhanen J, Lakka TA, Manninen P, Kaplan GA, Salonen JT (2005). Depression and early retirement: prospective population based study in middle aged men. J Epidemiol Community Health.

[7] Bültmann U, Rugulies R, Lund T, Christensen KB, Labriola M, Burr H (2006). Depressive symptoms and the risk of long-term sickness absence: a prospective study among 4747 employees in Denmark. Soc Psychiatry Psychiatr Epidemiol.

[8] Henderson M, Glozier N, Holland EK (2005). Long term sickness absence. Br Med J.

[9] Harvey SB, Glozier N, Henderson M, Allaway S, Litchfield P, Holland-Elliott K, Hotopf M (2011). Depression and work performance: an ecological study using web-based screening. Occup Med (Lond).

[10] Solomon C, Poole J, Palmer KT, Coggon D (2007). Health-related job loss: findings from a community-based survey. Occup Environ Med.

[11] Bhui KS, Dinos S, Stansfeld SA, White PD (2012). A synthesis of the evidence for managing stress at work: a review of the reviews reporting on anxiety, depression, and absenteeism. J Environ Public Health.

[12] Corbière M, Shen J, Rouleau M, Dewa CS (2009). A systematic review of preventive interventions regarding mental health issues in organizations. Work.

[13] Joyce S, Modini M, Christensen H, Mykletun A, Bryant R, Mitchell PB, Harvey SB (2016). Workplace interventions for common mental disorders: a systematic meta-review. Psychol Med.

[14] Vanhove AJ, Herian MN, Perez AL, Harms PD, Lester PB (2015). Can resilience be developed at work? A meta-analytic review of resilience-building programme effectiveness. J Occup Organ Psychol.

[15] Dewa CS, Hoch JS (2015). Barriers to mental health service use among workers with depression and work productivity. J Occup Environ Med.

[16] Lim D, Sanderson K, Andrews G (2000). Lost productivity among full-time workers with mental disorders. J Ment Health Policy Econ.

[17] Carolan S, Harris P, Cavanagh K (2017). Improving employee wellbeing and effectiveness: a systematic review and meta-analysis of workplace psychological interventions delivered online. J Med Internet Res.

[18] Ebert DD, Heber E, Berking M, Riper H, Cuijpers P, Funk B, Lehr D (2016). Self-guided internet-based and mobile-based stress management for employees: results of a randomised controlled trial. Occup Environ Med.

[19] Heber E, Lehr D, Ebert DD, Berking M, Riper H (2016). Web-based and mobile stress management intervention for employees: a randomized controlled trial. J Med Internet Res.

[20] Thiart H, Lehr D, Ebert DD, Berking M, Riper H (2015). Log in and breathe out: internet-based recovery training for sleepless employees with work-related strain - results of a randomized controlled trial. Scand J Work Environ Health.

[21] Umanodan R, Shimazu A, Minami M, Kawakami N (2014). Effects of computer-based stress management training on psychological well-being and work performance in japanese employees: a cluster randomized controlled trial. Ind Health.

[22] Cavanagh K, Millings A (2013). Increasing engagement with computerised cognitive behavioural therapies. ICST Trans Amb Sys.

[23] Griffiths F, Lindenmeyer A, Powell J, Lowe P, Thorogood M (2006). Why are health care interventions delivered over the internet? A systematic review of the published literature. J Med Internet Res.

[24] Spek V, Cuijpers P, Nyklícek I, Riper H, Keyzer J, Pop V (2007). Internet-based cognitive behaviour therapy for symptoms of depression and anxiety: a meta-analysis. Psychol Med.

[25] Junge M, Lehr D, Bockting C, Berking M, Riper H, Cuijpers P, Ebert D (2015). For whom are internet-based occupational mental health interventions effective? Moderators of internet-based problem-solving training outcome. Internet Interv.

[26] Waller R, Gilbody S (2009). Barriers to the uptake of computerized cognitive behavioural therapy: a systematic review of the quantitative and qualitative evidence. Psychol Med.

[27] de Visser RO, Graber R, Hart A, Abraham C, Scanlon T, Watten P, Memon A (2015). Using qualitative methods within a mixed-methods approach to developing and evaluating interventions to address harmful alcohol use among young people. Health Psychol.

[28] Carolan S, Harris PR, Greenwood K, Cavanagh K (2016). Increasing engagement with, and effectiveness of, an online CBT-based stress management intervention for employees through the use of an online facilitated bulletin board: study protocol for a pilot randomised controlled trial. Trials.

[29] Carolan S, Harris P, Greenwood K, Cavanagh K (2017). Increasing engagement with an occupational digital stress management program through the use of an online facilitated discussion group: results of a pilot randomised controlled trial. Internet Interv.

[30] Cohen S, Kamarck T, Mermelstein R (1983). A global measure of perceived stress. J Health Soc Behav.

[31] Jackson P, McKergow M (2002). The Solutions Focus: The SIMPLE Way to Positive Change.

[32] Braun V, Clarke V (2006). Using thematic analysis in psychology. Qual Res Psychol.

[33] Brouwer W, Oenema A, Crutzen R, de Nooijer J, de Vries NK, Brug J (2008). An exploration of factors related to dissemination of and exposure to internet-delivered behavior change interventions aimed at adults: a Delphi study approach. J Med Internet Res.

[34] Knowles S, Toms G, Sanders C, Bee P, Lovell K, Rennick-Egglestone S, Coyle D, Kennedy CM, Littlewood E, Kessler D, Gilbody S, Bower P (2014). Qualitative meta-synthesis of user experience of computerised therapy for depression and anxiety. PLoS One.

[35] Knowles SE, Lovell K, Bower P, Gilbody S, Littlewood E, Lester H (2015). Patient experience of computerised therapy for depression in primary care. BMJ Open.

[36] Ebert DD, Lehr D, Boß L, Riper H, Cuijpers P, Andersson G, Thiart H, Heber E, Berking M (2014). Efficacy of an internet-based problem-solving training for teachers: results of a randomized controlled trial. Scand J Work Environ Health.

[37] Lehr D, Geraedts A, Persson Asplund R, Khadjesari Z, Heber E, de Bloom J, Ebert DD, Angerer P, Funk B, Wiencke M, Cacace M, Fischer S (2016). Occupational e-Mental Health: Current Approaches and Promising Perspectives for Promoting Mental Health in Workers. Healthy at Work.

[38] Prochaska J, DiClemente C (1982). Transtheoretical therapy: toward a more integrative model of change. Psychotherapy.

[39] Prochaska JO, DiClemente CC, Norcross JC (1992). In search of how people change. Applications to addictive behaviors. Am Psychol.

[40] Krupnick JL, Sotsky SM, Simmens S, Moyer J, Elkin I, Watkins J, Pilkonis PA (1996). The role of the therapeutic alliance in psychotherapy and pharmacotherapy outcome: findings in the National Institute of Mental Health Treatment of Depression Collaborative Research Program. J Consult Clin Psychol.

[41] Clarke J, Proudfoot J, Whitton A, Birch M, Boyd M, Parker G, Manicavasagar V, Hadzi-Pavlovic D, Fogarty A (2016). Therapeutic alliance with a fully automated mobile phone and web-based intervention: secondary analysis of a randomized controlled trial. JMIR Ment Health.

[42] Alfonsson S, Olsson E, Hursti T (2016). Motivation and treatment credibility predicts dropout, treatment adherence, and clinical outcomes in an internet-based cognitive behavioral relaxation program: a randomized controlled trial. J Med Internet Res.

[43] Brouwer W, Oenema A, Crutzen R, de Nooijer J, de Vries N, Brug J (2009). What makes people decide to visit and use an internet‐delivered behavior‐change intervention?: A qualitative study among adults. Health Educ.

[44] Ludden GD, van Rompay TJ, Kelders SM, Van Gemert-Pijnen JE (2015). How to increase reach and adherence of web-based interventions: a design research viewpoint. J Med Internet Res.

[45] Bakker D, Kazantzis N, Rickwood D, Rickard N (2016). Mental health smartphone apps: review and evidence-based recommendations for future developments. JMIR Ment Health.

[46] Fry JP, Neff RA (2009). Periodic prompts and reminders in health promotion and health behavior interventions: systematic review. J Med Internet Res.

[47] Hilvert-Bruce Z, Rossouw PJ, Wong N, Sunderland M, Andrews G (2012). Adherence as a determinant of effectiveness of internet cognitive behavioural therapy for anxiety and depressive disorders. Behav Res Ther.

[48] Whitton AE, Proudfoot J, Clarke J, Birch M, Parker G, Manicavasagar V, Hadzi-Pavlovic D (2015). Breaking open the black box: isolating the most potent features of a web and mobile phone-based intervention for depression, anxiety, and stress. JMIR Ment Health.

[49] Wahle F, Bollhalder L, Kowatsch T, Fleisch E (2017). Toward the design of evidence-based mental health information systems for people with depression: a systematic literature review and meta-analysis. J Med Internet Res.

[50] Baldwin ML, Marcus SC (2007). Labor market outcomes of persons with mental disorders. Ind Relat.

[51] Krupa T, Kirsh B, Cockburn L, Gewurtz R (2009). Understanding the stigma of mental illness in employment. Work.

[52] Mearns K, Hope L, Ford MT, Tetrick LE (2010). Investment in workforce health: exploring the implications for workforce safety climate and commitment. Accid Anal Prev.

[53] Abbott JM, Kaldo V, Klein B, Austin D, Hamilton C, Piterman L, Williams B, Andersson G (2009). A cluster randomised trial of an internet-based intervention program for tinnitus distress in an industrial setting. Cogn Behav Ther.

[54] Abbott J, Klein B, Hamilton C, Rosenthal AJ (2009). The impact of online resilience training for sales managers on wellbeing and performance. E-JAP.

[55] Persson AR, Dagöö J, Fjellström I, Niemi L, Hansson K, Zeraati F, Ziuzina M, Geraedts A, Ljótsson B, Carlbring P, Andersson G (2017). Internet-based stress management for distressed managers: results from a randomised controlled trial. Occup Environ Med.

[56] Geraedts A, Kleiboer A, Wiezer N, Cuijpers P, van Mechelen W, Anema J (2014). Feasibility of a worker-directed web-based intervention for employees with depressive symptoms. Internet Interv.

[57] Ebert DD, Lehr D, Smit F, Zarski A, Riper H, Heber E, Cuijpers P, Berking M (2014). Efficacy and cost-effectiveness of minimal guided and unguided internet-based mobile supported stress-management in employees with occupational stress: a three-armed randomised controlled trial. BMC Public Health.

[58] Lui JH, Marcus DK, Barry CT (2017). Evidence-based apps? A review of mental health mobile applications in a psychotherapy context. Prof Psychol Res Pr.

[59] Johansson R, Sjöberg E, Sjögren M, Johnsson E, Carlbring P, Andersson T, Rousseau A, Andersson G (2012). Tailored vs. standardized internet-based cognitive behavior therapy for depression and comorbid symptoms: a randomized controlled trial. PLoS One.

[60] Andersson G, Cuijpers P (2009). Internet-based and other computerized psychological treatments for adult depression: a meta-analysis. Cogn Behav Ther.

[61] Richards D, Richardson T (2012). Computer-based psychological treatments for depression: a systematic review and meta-analysis. Clin Psychol Rev.

[62] Heber E, Ebert DD, Lehr D, Cuijpers P, Berking M, Nobis S, Riper H (2017). The benefits of web- and computer-based interventions for stress: a systematic review and meta-analysis. J Med Internet Res.

[63] Baumeister H, Reichler L, Munzinger M, Lin J (2014). The impact of guidance on Internet-based mental health interventions — a systematic review. Internet Interv.

[64] Humphreys K, Marx B, Lexington J, O'Donohue WT, Fisher JE (2009). Self Monitoring as a Treatment Vehicle. General Principles and Empirically Supported Techniques of Cognitive Behaviour Therapy.

[65] Griffiths KM, Calear AL, Banfield M (2009). Systematic review on Internet Support Groups (ISGs) and depression (1): do ISGs reduce depressive symptoms?. J Med Internet Res.

[66] Melling B, Houguet-Pincham T (2011). Online peer support for individuals with depression: a summary of current research and future considerations. Psychiatr Rehabil J.

[67] Ali K, Farrer L, Gulliver A, Griffiths KM (2015). Online peer-to-peer support for young people with mental health problems: a systematic review. JMIR Ment Health.

